# Discoverability of African Journals by Google Scholar and Inclusion in Scopus

**DOI:** 10.12688/gatesopenres.16372.1

**Published:** 2025-11-18

**Authors:** Patrick Amboka, Julius Kirimi Sindi, Marylene Wamukoya, Nosa Orobaton, Alphonsus Neba, Marta Vicente-Crespo, Evelyn Gitau

**Affiliations:** 1 African Population and Health Research Center, Nairobi, Nairobi County, Kenya; 2 Bill & Melinda Gates Foundation, Seattle, Washington, USA; 3 University of the Witwatersrand Johannesburg School of Public Health, Johannesburg, Gauteng, South Africa; 4 Science For Africa Foundation, Nairobi, Nairobi County, Kenya

**Keywords:** Journal indexing, international standards, discoverability, African Journals, Capacity strengthening, Research evaluation

## Abstract

**Background:**

There has been steady progress and advancement of research in Africa. However, African researchers face numerous challenges among them, limited international recognition. This is due to the low discoverability and inclusion of their research outputs by indexers and databases. A lot of initiatives have attempted to address the challenge, however, there is a need for support to enhance the discoverability and inclusion of research outputs from Africa.

**Methods:**

We conducted a desk review of 1,116 journals hosted on the Sabinet journal repository and the African Journal Online (AJOL) platform. The factors that were considered to influence journals’ discoverability and inclusion include (i) the journals’ Open Access (OA) status, (ii) OA journals’ listing in the Directory of Open Access Journals (DOAJ), (iii) the journals’ presence on the International Standard Serial Number (ISSN) portal, (iv) the membership of the journals’ publishers on the Committee on Publication Ethics (COPE), (v) the journals’ hosting on International Network for Advancing Science and Policy (INASP) and (vi) geographic location of the journals’ online publisher.

**Findings:**

A total of 1,116 journals were identified from the Sabinet and AJOL platforms. The highest proportion of journals was neither discovered by Google Scholar nor included in Scopus (63.2%). The study established one significant predictor of journal discoverability by Google Scholar and inclusion in Scopus. This was the journal listing on the ISSN portal which increased the odds of the journal being discoverable by Google Scholar and inclusion in Scopus by 2.033 and 5.451 respectively. Journals listed in the DOAJ but whose publishers were COPE members had significantly reduced odds of being discoverable by Google Scholar and being included in Scopus by 0.334 and 0.161 respectively. This suggests that the journal’s discoverability and inclusion are more nuanced and not always straightforward hence quality markers need to be aligned.

## Introduction

African researchers contribute to the advancement of knowledge in their fields and provide important insights into the issues affecting their communities. African Voices in creativity and learning, as researchers and interlocutors in relation to various global health institutions/policies, are critical despite the legacies of colonial research and practices. ^1^ Despite the percentage of Sub-Saharan Africa (SSA) share of global research increasing from 0.44% to 0.72% between 2003-2012, the region still accounts for less than one percent of the world’s research output. ^2^ African scientific research outputs are not adequately discoverable on international platforms and the internet at large since they are not indexed. ^3^ African researchers as keen contributors to knowledge as a whole and as a global public good. ^1^ However, they still face the challenge of limited international recognition by the subject matter experts. ^4^ The failure of international recognition is a key contributor to the research inequalities which are more pronounced in social sciences, humanities, arts, and related fields. ^5^ Many African researchers publish in journals that are hosted in local repositories, based at universities or institutions. ^6^ African researcher opts to publish to their institutional repositories due to the absence of connections to international databases because of financial constraints. The performance of African institutional repositories remains below average thus making the African researchers’ output not discoverable since they can’t be connected to international repositories. ^7^ The reasons for this include language barriers and limited funding that reduces the scope of the studies as well as their capacity to do cutting-edge research. In addition, non-white researchers are underrepresented in editorial boards relative to the expected share by their quota of authorship and are less likely to be invited to review articles for journals, even after controlling for factors such as publication records and academic affiliation. ^8– 12^ This has an impact beyond the representation *per se* since editors act as “opinion formers, gatekeepers, and arbiters of disciplinary values” ^13, 14^ and studies have shown the existence of bias against non-English names in education assessment, evaluation of skill sets, and the peer-review process. ^15^ Thus, African journals stand to play an important role in mitigating the aforementioned factors and improving their discoverability could lead to increased awareness of important health information, improved health outcomes, and more robust scientific discourse that is representative of the continent’s contexts. In this study, we aim to map and describe the current state of discoverability of African journals on international platforms and determine factors associated with their discoverability.

Journal indexing is being used to add journals to databases or indices to facilitate searching and retrieving information about academic journals, their contents, and their content producers. Journal indexing is important to the reach, reputation, and impact of journal articles since many scholars prefer submitting articles to journals indexed by leading indexers as they are perceived to be of good quality. ^16^ Journal indexing services typically use a set of criteria to evaluate the quality and relevance of a journal’s content for a database and/or index and such criteria include the peer-review process, citation count, and impact factor. ^17^ Journals that are indexed are perceived to be of higher quality and receive a higher number of citations hence more visibility. ^18^ The more the journal is indexed in many databases, the more it becomes discoverable. ^16^

Various platforms/systems have been set up to increase journal access and discoverability. They include Google Scholar (GS) platform, Scopus platform, Open Access (OA) system, Directory of Open Access Journals (DOAJ) platform, International Standard Serial Number (ISSN) platform, International Network for Advancing Science and Policy (INASP) platform, Web of Science (WoS) platform and PubMed platform. Over time, several discovery services have emerged but there isn’t one that indexes all the journals that exist. ^19^ The Scopus is an internationally recognized scientific database that provides valuable insights into the academic landscape ^13^ by providing comprehensive and reliable information to increase the discoverability of academic research and ensure that it is widely recognized and utilized within the academic community. ^20^ While Scopus is not free, Google Scholar is a free search engine ^21^ that is most widely used for both non-academic and academic content. ^22^ This search engine offers the most comprehensive coverage, making it ideal for finding research outputs and valuable information for evaluating research products published in journals from developing countries. ^23^

A journal’s inclusion in these databases and indices depends on various factors. They include: (i) the journal’s open access status (OA), (ii) the journal being listed in the directory of open access (DOAJ), (iii) the journal’s presence on the International Standard Serial Number (ISSN) portal, (iv) the journal publisher being a member of the Committee on Publication Ethics (COPE), (v) the journal being hosted on the International Network for Advancing Science and Policy (INASP) and (vi) the journal publisher’s geographic location.

OA journals provide unrestricted online access to their content and their articles are made freely available online without any financial, legal, or technical barriers ^24^ and are available to anyone with an internet connection to read, download, copy, distribute, print, or search. These bridge the digital divide by providing equal access to information and knowledge for researchers in Africa and other low-income countries (LICs) and also inadvertently, have a higher number of citations. ^25^ However, these OA journals still require publication fees and these can be a barrier to publishing here. ^24^

The Directory of Open Access (DOAJ) is a community-curated online directory that indexes high-quality, peer-reviewed OA scholarly journals to make them accessible. It is important to note that these often have inclusion criteria that must be met, that is, the journal must provide sufficient information about itself and the publication process and it must adhere to best practices of OA publishing that have been agreed upon, namely licensing and copyright policies. ^26^ DOAJ uses inclusion criteria which include ease of identifying contact and identifying contact and identity of the publisher, clarity on the type of peer review, whether the article within the journal is archived/indexed, fees charged, guidelines provided for authors on the website of the publisher, the journal publisher’s membership of a recognized industry initiative such as the Committee on Publication Ethics (COPE).

An International Standard Serial Number (ISSN) is a unique identifier assigned to a serial publication, such as a journal, magazine, or newspaper. This number is an eight-digit code that is used to identify the publication to distinguish it from other publications, to improve the discoverability and management of serial publications ^27^ and to facilitate the organization and retrieval of serial publications. ^28^ It is widely recognized as an essential tool for librarians, publishers, and researchers. To receive an ISSN, a journal must provide basic information about itself (its title, its publisher, its editorial board, and its frequency of publishing new editions) and is then placed on the ISSN’s portal. Furthermore, all journals present on the portal adhere to the standards set by the ISSN International Centre, which include publishing the journal regularly, using a consistent title, and providing accurate bibliographic information. ^29^

The Committee on Publication Ethics (COPE) is a non-profit organization that provides guidance and support to publishers, editors, and authors in the conduct of ethical research and publication practices. COPE has been recognized for its role in promoting ethical standards in academic publishing, including its impact on the discoverability of research. By providing guidance and best practices for authors, editors, and publishers, COPE helps to ensure that the published work is of high quality and is free of ethical misconduct. ^30^ Journal publishers become COPE members by agreeing to the COPE code of conduct, which includes following ethical guidelines for research and publication, maintaining editorial independence, and ensuring the quality and integrity of the journal. ^30^

The International Network for Advancing Science and Policy (INASP) is a non-profit organization that works to strengthen the capacity of individuals and institutions in low and middle-income countries (LMICs) to produce, share, and use research and knowledge for sustainable development. The network curates a list of journals and provides information about journals’ editorial policies, peer-review processes, and publication standards and their efforts have helped to ensure that research produced in LMICs is widely recognized and cited, thereby addressing the “knowledge gap” between LMICs and other countries. ^31^ For journals to be hosted on INASP, they must be indexed in recognized databases, such as Scopus, WoS, or PubMed, and obtain a Digital Object Identifier (DOI), which can be a challenge for journals with limited resources. ^32^ INASP uses the Journal Publishing Practices and Standards (JPPS) to assess journals for inclusion. The standards include; whether the journal has met basic requirements for at least two years (one star), whether the journal is compliant with the quality criteria of editorial publishing practice (two stars), the consistency of the journal in all technical and editorial publishing best practices of the JPPS (three stars).

Journal publishing in Africa has been growing in recent years and it is important to harness the increase in the number of African-based publishers emerging in the continent’s research arena. ^19^ There must, therefore, be concomitant efforts to increase the discoverability and accessibility of research produced on the continent and to promote the use of African research in decision-making processes. Journal publishers based in Africa are key to the development of a vibrant research community in the region and have enabled endeavors to ensure that the knowledge produced in Africa is widely recognized and used. ^33^

This study examined the factors associated with the discoverability of African research by, Google Scholar and inclusion in Scopus to characterize the current state of discoverability/inclusion.

The two platforms differ in how they enhance the discoverability and inclusion of academic content, as well as in the types of content they support. Scopus is an internationally recognized scientific database that requires a subscription to access comprehensive and reliable information included in it. On the other hand, Google Scholar is a free search engine that is most widely used for both non-academic and academic content. ^22^ Additionally, Google Scholar is a crawler-based search engine unlike Scopus. ^16^ Google Scholar discovers and indexes academic research by discovering the content using the search engine. These two indexers focus on all disciplines unlike an indexer like PubMed which has the primary focus on life sciences and biomedical sciences. ^16^ Google Scholar and Scopus are currently the most comprehensive search engines in varied disciplines. ^21^ This information is vital for ameliorating the discoverability of African research and producers and of African research to ensure that efforts that are geared towards regional agendas are contextual and relevant to African society.

## Methods

The methods section development followed the Enhancing the Quality and Transparency of Health Research (EQUATOR) reporting to improve reproducibility and ensure validity. ^34^

### Data provider

Data on journals title extract form AJOL and Sabinet African journal repositories. Data about journals extract from the journals’ websites, the DOAJ platform, the ISSN portal, COPE website, and INASP platform.

### Search strategy

The search to identify the journals was performed in March 2023. The search strategy focused on the following inclusion criteria: (i) that a journal was hosted on one of two major collections, the Africa Journals Online (AJOL) platform ^35^ and the Sabinet African Journals repository, ^36^ and (ii) that the journal was hosted in an African institution, regardless of the publisher’s nationality and/or physical location. Journals meeting this set of criteria were included regardless of whether the journal was actively publishing or not at the time of the study. Any journal that met the aforementioned criteria was included in the study, although we took a cautious approach to avoid inadvertently excluding any journals.

### Sifting and sorting (Data cleaning)

The titles of the journals were carefully noted to remove duplicates. We counter-checked the publishers of all duplicate journals and maintained only one record where the publishers’ names were identical.

### Data collection/extraction

A data collection tool was created to capture detailed data from the journals in the Sabinet repository and the AJOL platform. Through this tool, we collected information on the following:

• Journal status indexing on Google Scholar ^37^
• Journal status indexing on Scopus ^38^
• OA status of the journal
• OA journal is listed in the DOAJ ^26^
• Presence on the ISSN portal ^39^
• Journal publisher’s COPE membership ^40^
• Journal presence on INASP listing ^41^
• Journal publisher’s geographical location

We searched the journals’ websites to find out the OA status and the publisher’s geographical location. We searched each of the journals’ names in the DOAJ and INASP platforms, the ISSN portal. We searched for the publisher’s name on the COPE website.

To determine the discoverability of the journals by Google Scholar, we searched for the names of the journals using the search tool under the Metrics section of Google Scholar. ^37^ When a journal is discoverable by Google Scholar, this search results in a hit including the name of the journal, the h5-index, and the h5-median.

To determine if a journal is included in the Scopus database, we searched the Scimago Journal Rank. ^38^ When a journal is indexed in Scopus, this search results in a hit with the journal’s name. Further information can be found in the file, but the variables were out of the scope of this study.

### Data extraction date (DED)

Data extraction for this study was performed between 1 ^st^ April 2023 and 30 ^th^ June 2023.

### Sampling strategy

Sampling did not apply to this study because all the source data (Journals hosted on AJOL and Sabinet journal repositories) was utilized.

### Source data range (SDR)

Source data available and updated on the platforms between 1 ^st^ April 2023 and 30 ^th^ June 2023 was extracted.

### Journal listing, discoverability or inclusion window

The study focused on the data available on the platform and there was no specific period the variables were required to have been discoverable or included in the source data. The study focused on the current status of the journals during data extraction.

### Data processing

• Data was then processed using Stata version 17.0. To ensure that our variables of analysis had binary codes as follows: The dependent variable was created as follows:
  ○ A variable was created to indicate if a journal was discoverable by Google Scholar, and it was assigned the code ‘1’ to indicate ‘Yes – the journal was discoverable by Google Scholar’ otherwise it was assigned ‘0’ to indicate ‘No’
  ○ A variable was created to indicate if a journal was included in Scopus, and it was assigned the code ‘1’ to indicate ‘Yes – the journal was on Scopus’ otherwise it was assigned ‘0’ to indicate ‘No’
  ○ Subsequently, a dependent variable was created to denote a journal’s visibility by combining the above two variables to indicate if a journal was discoverable by Google Scholar and included in Scopus was assigned the code ‘1’ to indicate ‘Yes – the journal was discoverable by Google scholar and included in Scopus’ and otherwise it was assigned ‘0’ to indicate ‘No’
  ○ A dependent variable was also created to denote a journal’s discoverability/inclusion by combining the above two variables to indicate if a journal was discoverable/included in at least one of the platforms. The journal was assigned the code ‘1’ to indicate ‘Yes – the journal was discoverable/included in at least one platform, otherwise it was assigned ‘0’ to indicate ‘No’
• The independent variables (predictors) did not have missing values, and all the desired information was readily available. These binary variables were created as follows:
  ○ OA status of the journal - coded ‘1’ to indicate ‘Yes – the journal was open access’ and otherwise coded ‘0’ to indicate ‘No’
  ○ OA journal is listed in the DOAJ - coded ‘1’ to indicate ‘Yes – that the OA journal was listed on the DOAJ’ and otherwise coded ‘0’ to indicate ‘No’
  ○ Presence on the ISSN portal - coded ‘1’ to indicate ‘Yes – the journal was present on the ISSN portal’ and otherwise coded ‘0’ to indicate ‘No’
  ○ Journal publisher’s COPE membership - coded ‘1’ to indicate ‘Yes – the journal publisher was a member of COPE’ and otherwise coded ‘0’ to indicate ‘No’
  ○ Journal presence on INASP listing - coded ‘1’ to indicate ‘Yes – the journal was listed on INASP’ and otherwise coded ‘0’ to indicate ‘No’
  ○ Journal publisher’s geographical location - coded ‘1’ to indicate ‘Yes – the journal publisher was based in Africa and otherwise coded ‘0’ to indicate ‘No’

### Data analysis

This was conducted in Stata version 17.0. As all the variables were categorical, they were analyzed using the χ ^2^ (Chi-square) test to obtain a crude odd ratio (COR) with confidence intervals (CI) of 95%. All statistical tests were 2-sided, and a p-value of less than 0.05 was considered to indicate statistical significance. Multivariate logistic regression models were used to understand the association between a journal’s discoverability by Google Scholar and inclusion in the Scopus database. The logistic regression predicted the odds of a journal being discoverable by Google Scholar and being included in the Scopus database based on these independent variables: OA status of the journal, OA journal is listed in the DOAJ, presence on the ISSN portal, journal publisher’s COPE membership, journal presence on INASP platform and the journal publisher’s geographical location. The resulting odds ratios (OR) were used to understand these associations as follows: an OR of 1 reflected that there was no association between the independent factor and being discoverable/included in Google Scholar and/or Scopus, an OR between 0 and 1 meant that the independent factor had negative effects, where a one-unit increase in the factor decreased the odds of being discoverable and decreased the odds and an OR that was greater than 1 showed that the independent factor had positive effects as a one-unit increase in the factor increased the odds of being discoverable/included. Marginal effects (ME) were computed to understand how much a unit change in each independent variable changed the probability of a journal being visible (holding all the other independent variables constant). Since the independent variables were binary, the marginal effects measured discrete change, that is, how much and in which direction the probability of a journal’s visibility changed when the independent variable changed from ‘0’ to ‘1’. Comparisons between the groups were adjusted to remove confounders. A chunk test was done on all the models comparing the models with interactions and the models without interactions. The Chunk test evaluated the hypothesis that there are no significant terms within our models. A P-value of <0.05 indicated that the model with interaction term(s) was a better fit. Conversely, a higher P-value suggested the opposite.

## Results

### Journals identified in AJOL and Sabinet repositories

**Figure 1.**
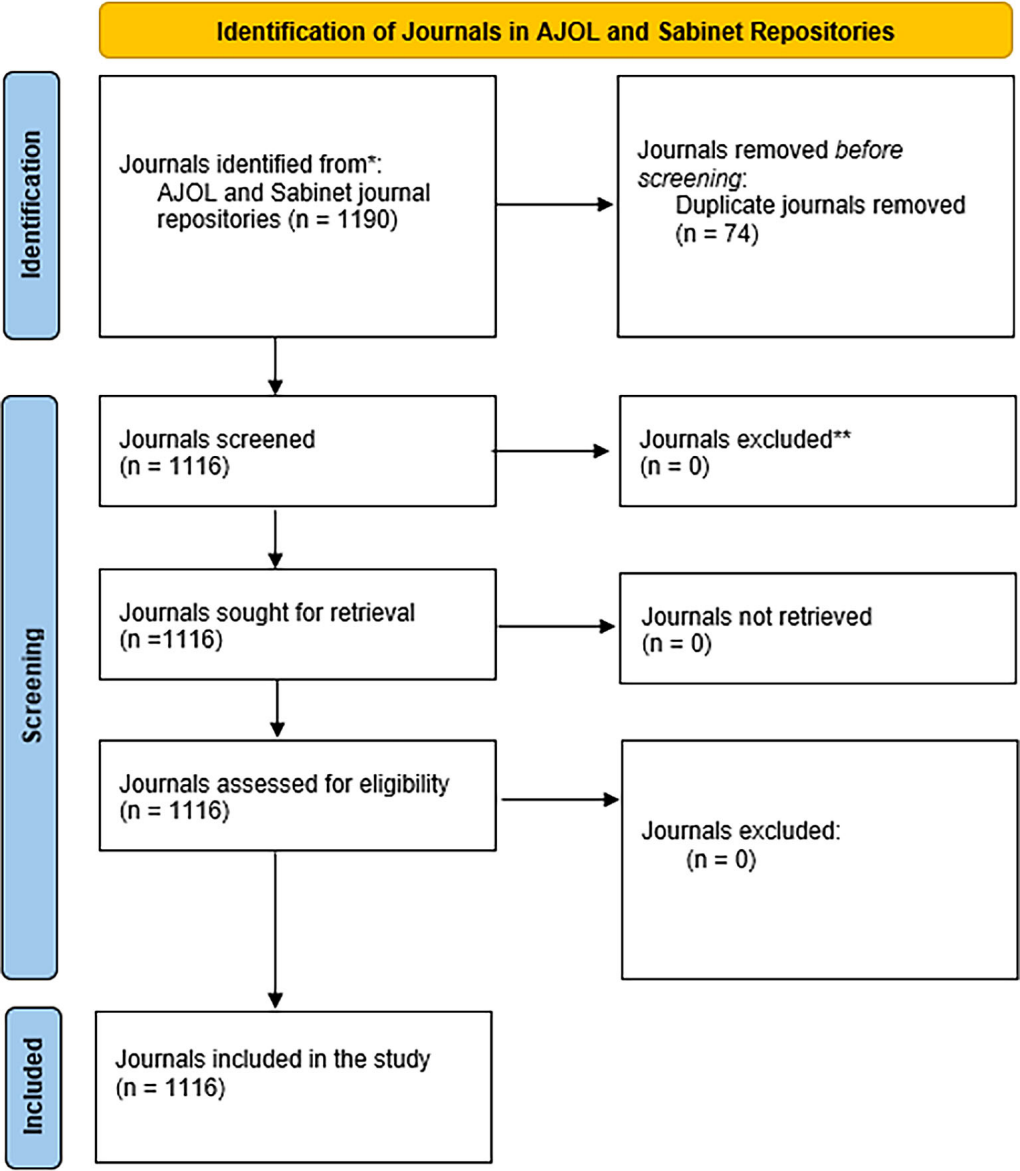
The process of identifying journals.

A total of 1,190 journals were identified and retrieved from the AJOL platform and the Sabinet repository during the search period (March 2023). A total of 74 duplicates were removed, and analysis was conducted using data about the remaining 1,116 journals, as shown in ( Figure 1). All the journals identified were hosted by African institutions.

### Journals Discoverable by Google Scholar and included in Scopus Database

Of the 1,116 journals, slightly more than one-tenth (11.8%) were discoverable by Google Scholar and included the Scopus database whereas the majority (63.1%) were not discoverable/included on either one of the two platforms ( Table 1). Close to one-fifth (19.9%) of the journals were discoverable by Google Scholar only and 5.1% were included in Scopus only. When disaggregated by the various independent variables, journals that were discoverable by Google Scholar only were of a larger proportion compared to journals that were included in Scopus only. Specifically, of those journals that were OA (418) or those that were present on INASP listing (580), or those where the publisher was based in Africa (1,015), about one-fifth (21.3%, 19.7% and 19.9%, respectively) were discoverable by Google scholar only while less than 5% (4.3%, 4.7% and 4.2%, respectively) were included in Scopus only. Of the OA journals, 104 were listed in the DOAJ, and while almost one-quarter of these (23.1%) were discoverable by Google Scholar only 6.7% were included in Scopus only. Just more than one-fifth (21.3%) of the 825 journals that were present on the ISSN portal were discoverable by Google Scholar only while only 6.6% were included in Scopus only. A journal publisher’s membership in COPE was evidenced in only 129 of these journals with 19.4% being discoverable by Google Scholar only and 17.0% being included in Scopus only. The results in Table 2 indicate that the majority of journals were non-open access (NOA) and accounted for 62.5% of the journals. Of the 418 OA journals, 35.9% were discoverable by Google Scholar, and 23.3% were included in Scopus while among NOA journals 29.3% were discoverable by Google Scholar, and 15.8% were included in Scopus. The association between OA status and Google Scholar discoverability was significant with a COR of 1.35 [p-value=0.02]. However, the association between OA status and Scopus inclusion was insignificant [COR=1.25; p-value=0.18].

**Table 1.** Journals discoverable/included in either Google Scholar only, Scopus only, both Google Scholar and Scopus, or none of them.

	Total	Discoverable by Google Scholar and included in Scopus	Discoverable by only Google Scholar	Included in only Scopus	Discoverable/included in none	P-value
Total number of journals	1,116	132 (11.8%)	222 (19.9%)	57 (5.1%)	705 (63.1%)	
OA journals	418	61 (14.6%)	89 (21.3%)	18 (4.3%)	250 (59.8%)	0.07
NOA journals	698	71 (10.2%)	133 (19.1%)	39 (5.6%)	455 (65.2%)	
OA journals listed in DOAJ	104	41 (39.4%)	24 (23.1%)	7 (6.7%)	32 (30.8%)	<0.001
OA journals not listed in DOAJ	314	20 (6.4%)	65 (20.7%)	11 (3.5%)	218 (69.4%)	
Journals present on the ISSN portal	825	127 (15.4%)	176 (21.3%)	54 (6.6%)	468 (56.7%)	<0.001
Journals absent on the ISSN portal	291	5 (1.7%)	46 (15.8%)	3 (1.0%)	237 (81.4%)	
Journals whose publisher is a member of COPE	129	54 (41.9%)	25 (19.4%)	22 (17.0%)	2 8(21.7%)	<0.001
Journals whose publisher is not a member of COPE	987	78 (7.9%)	197 (20.0%)	35 (3.6%)	677 (68.6%)	
Journals present on the INASP listing	580	80 (13.8%)	114 (19.7%)	27 (4.7%)	359 (61.9%)	0.36
Journals absent on the INASP listing	536	52 (9.7%)	10 8(20.2%)	30 (5.6%)	346 (64.6%)	
Journals publisher based in Africa	1,015	95 (9.4%)	202 (19.9%)	43 (4.2%)	675 (66.5%)	<0.001
Journals publisher based outside of Africa	101	37 (36.6%)	20 (19.8%)	14 (13.9%)	30 (29.7%)	

OA: Open access; NOA: Not open access; DOAJ: Directorate of Open Access Journals; ISSN: International Standard Serial Number; COPE: Committee on Publication Ethics; INASP: International Network for Advancing Science and Policy.

**Table 2.** Factors associated with journal discoverability by Google Scholar and included in Scopus.

	Discoverable by Google Scholar	Not discoverable by Google Scholar	Total	COR	P-value	Included in Scopus	Not included in Scopus	Total	COR	P-value
OA journals	150 (35.9%)	268 (64.1%)	418	1.35	0.02	79 (23.3%)	339 (76.7%)	418	1.25	0.18
NOA journals	204 (29.3%)	494 (70.8%)	698			110 (15.8%)	588 (84.2%)	698		
Total	354	762	1,116			189	927	1,116		
OA journals listed in DOAJ	65 (62.5%)	39 (37.5%)	104	4.49	<0.001	48 (46.2%)	56 (53.9%)	104	7.83	<0.001
OA journals unlisted in DOAJ	85 (27.1%)	229 (72.9%)	314			31 (9.9%)	283 (90.1%)	314		
Total OA journals	150	268	418			79	339	418		
Total NOA journals	204	494	698			110	588	698		
Total	354	762	1,116			189	927	1,116		
Journals present on the ISSN portal	303 (36.7%)	522 (63.3%)	825	2.73	<0.001	181 (21.9%)	644 (78.1%)	825	9.94	<0.001
Journals absent on the ISSN portal	51 (17.5%)	240 (82.5%)	291			8 (2.8%)	283 (97.3%)	291		
Total	354	762	1,116			189	927	1,116		
Journals whose publisher was a member of COPE	79 (61.2%)	50 (38.8%)	129	4.09	<0.001	76 (58.9%)	53 (41.1%)	129	11.09	<0.001
Journals whose publisher was not a member of COPE	275 (27.9%)	712 (72.1)	987			113 (11.5%)	874 (88.6%)	987		
Total	334	762	1,116			189	927	1,116		
Journals present on the INASP listing	194 (33.5%)	386 (66.6%)	580	1.18	0.10	107 (18.5%)	473 (81.6%)	580	1.25	0.16
Journals absent on the INASP listing	160 (29.9%)	376 (70.2%)	536			82 (15.3%)	454 (84.7%)	536		
Total	354	762	1,116			189	927	1,116		
Publisher based in Africa	297 (29.3%)	718 (70.7%)	1,015	0.32	<0.001	138 (13.6%)	877 (86.4%)	1,015	0.15	<0.001
Publisher not based in Africa	57 (56.4%)	44 (43.6%)	101			51 (50.5%)	5 0 (49.5%)	101		
Total	354	762	1,116			189	927	1,116		

OA: Open access; NOA: Not open access; DOAJ: Directorate of Open Access Journals; ISSN: International Standard Serial Number; COPE: Committee on Publication Ethics; INASP: International Network for Advancing Science and Policy.

### OA journal is listed in the DOAJ

Of the 418 OA journals, only about one-tenth (9.32%) were listed on the DOAJ. A larger proportion were discoverable by Google Scholar than inclusion in Scopus (62.5% versus 46.2%, respectively). OA journals listed in DOAJ were about 5 times more likely to be discoverable by Google Scholar [COR=4.49; p-value<0.001] and 7.83 times more likely to be included in Scopus [p-value<0.001].

### Presence on the ISSN portal

Over three-quarters of the journals were listed on the ISSN portal (73.9%) as shown in Table 2. There were 303 journals discoverable by Google Scholar compared to 181. Journals that were present on the ISSN portal were 2.73 [p-value<0.001] times more likely to be discovered by Google Scholar almost ten times [COR=9.94; p-value<0.001] more likely to be included in Scopus.

### Journal publisher’s COPE membership

There were 129 journals whose publishers were members of COPE ( Table 2). The majority of journals (88.4%) did not have a publisher with this membership. On discoverability by Google Scholar, about two-thirds were discoverable (61.2%), and inclusion in Scopus (58.9%). The association between a journal having a publisher in COPE and being discoverable was significant for both Google Scholar [COR=4.09; p-value<0.001] and Scopus [COR=11.09; p-value<0.001].

### Journal presence on INASP listing

In Table 2, it is observed that about half (52.0%) of the journals were on the INASP listing. However, only one-third (33.5%) of these were discoverable by Google Scholar while just less than one-fifth (18.5%) were included in Scopus. There was no significant association between a journal being listed on INASP and being discoverable by Google Scholar [COR=1.18; p-value=0.01], and inclusion in Scopus [COR=1.25; p-value=0.16].

### Journal publisher’s geographical location

Of the 1,116 journals, the majority (91.0%) had their publisher based in Africa as shown in Table 2. There were differences in discoverability by Google Scholar versus inclusion in Scopus. Almost one-third (29.3%) of journals whose publisher was based in Africa were discoverable by Google Scholar. However, slightly above one-tenth (13.6%) of journals that had a publisher based in Africa were included in Scopus. When a publisher was based in Africa, the journal was less likely to be discoverable by Google Scholar [COR=0.32; p-value<0.001] or included in Scopus [COR=0.15; p-value<0.001].

### Journals discoverable by either Google Scholar or included in Scopus or none of the platforms

We found associations between several independent variables and whether or not a journal was included and discoverable on any of the platforms as seen in Table 3. Over one-third (36.8%) of the journals were discoverable/included on at least one of the platforms. Journals were significantly more likely to be discoverable/included on at least one of the platforms if they were OA [COR=1.26; p-value=0.07], were listed in the DOAJ [COR=5.11; p-value<0.001], were presented on the ISSN portal [COR=3.35; p-value<0.001] or had a publisher who was a member of COPE [COR=7.88; p-value<0.001]. However, those journals that had a publisher based in Africa [COR=0.21; p-value<0.001] were less likely to be discoverable/included in any of the platforms. The association between being present on the INASP listing and being discoverable/included in at least one platform was insignificant [COR=1.12; p-value=0.36].

**Table 3.** Factors associated with journal discoverability/inclusion in at least one platform (Google Scholar or Scopus).

	Discoverable/included in at least one platform	Discoverable/included in none of the platforms	Total	COR	P-value
OA journals	168 (40.2%)	250 (59.8%)	418	1.26	0.07
NOA journals	243 (34.8%)	455 (65.2%)	698		
Total	411	705	1,116		
OA journals listed in DOAJ	72 (69.2%)	32 (30.8%)	104	5.11	<0.001
OA journals not listed in DOAJ	96 (30.6%)	218 (69.4%)	314		
Total	168	250	418		
Journals present on the ISSN portal	357 (43.3%)	468 (56.7%)	825	3.35	<0.001
Journals absent from the ISSN portal	54 (18.6%)	237 (81.4%)	291		
Total	411	705	1,116		
Journals whose publisher was a member of COPE	101 (78.3%)	28 (21.7%)	129	7.88	<0.001
Journals whose publisher was not a member of COPE	310 (31.4%)	677 (68.6%)	987		
Total	411	705	1,116		
Journals present on the INASP listing	221 (38.1%)	359 (61.9%)	580	1.12	0.36
Journals absent on the INASP listing	190 (35.5%)	346 (64.6%)	536		
Total	411	705	1,116		
Publisher based in Africa	340 (33.5%)	675 (66.5%)	1,015	0.21	<0.001
Publisher not based in Africa	71 (70.3%)	30 (29.7%)	101		
Total	411	705	1,116		

OA: Open access; NOA: Not open access; DOAJ: Directorate of Open Access Journals; ISSN: International Standard Serial Number; COPE: Committee on Publication Ethics; INASP: International Network for Advancing Science and Policy; COR: Crude Odds Ratio.

### Multivariate analysis of factors associated with the discoverability of journals by Google Scholar

The collinearity test showed that two relevant interaction terms associated with the discoverability of journals by Google Scholar had low collinearity. Therefore, they were included in the model. The terms were: (1) interaction between OA journals listed in the DOAJ and Journal presence on the ISSN portal; and (2) interaction between journals listed in the DOAJ and the publishers being members of COPE. The two interaction terms were included in a multivariate logistic regression model. The regression models including and excluding the interaction terms were different as per the Chunk test [P-value: <0.05]. Thus, we selected the model including the interaction terms for further analysis since there were significant interaction terms within our model.

The model also showed that the journal presence on the ISSN portal significantly increased the discoverability by Google Scholar since the odds of being discoverable by Google Scholar were 2.033 [AOR=2.033; ME=0.137; p-value <0.001]. The effect of COPE membership on the journal’s discoverability by Google Scholar varied depending on whether the journal was listed in the DOAJ. Journals listed in the DOAJ but whose publishers were COPE members had significantly reduced odds of being discoverable by Google Scholar (about 33% of the odds compared to those without this combination) [AOR=0.334; ME=-0.212] as shown in Table 4. The logistic regression with interaction terms showed that when journals are listed in the DOAJ and their publishers are members of COPE, there is a significant interaction that leads to reduced discoverability by Google Scholar [P-value: <0.05]. OA, journal listing on the INASP platform, and publisher’s geographic location were insignificant predictors of discoverability by Google Scholar.

**Table 4.** Results from logistic regression of factors associated with journal discoverability by Google Scholar.

Variables	AOR	Robust Standard errors	Sig ^1^	Average Marginal Effects (ME)	Sig ^1^
OA journal	0.995	0.167	n/s	-0.001	n/s
Journals listed in DOAJ	7.690	7.186	*	0.394	*
Journals present on the ISSN portal	2.033	0.372	***	0.137	***
Journals whose publisher was a member of COPE	3.445	0.999	***	0.239	***
Journals present on the INASP listing	0.984	0.149	n/s	-0.003	n/s
Publisher based in Africa	0.816	0.950	n/s	-0.030	n/s
Interaction between OA journals listed in the DOAJ and Journal presence on the ISSN portal	**0.528**	**0.507**	**n/s**	**-0.123**	**n/s**
Interaction between journals listed in the DOAJ and the publisher being a member of COPE	**0.334**	**0.182**	*	-0.212	*
Constant	0.233	0.081	***		
Number of Observations	1116				
Pseudo R ^2^	0.08				

OA: Open access; NOA: Not open access; DOAJ: Directorate of Open Access Journals; ISSN: International Standard Serial Number; COPE: Committee on Publication Ethics; INASP: International Network for Advancing Science and Policy; AOR: Adjusted Odds Ratio.

^1^ Statistically significant is indicated by level, that is,

* p<0.05,

** p<0.01,

*** p<0.001 and n/s=not significant.

### Multivariate analysis of factors associated with the inclusion of journals in Scopus

The collinearity test showed that one relevant interaction term associated with the inclusion of journals in Scopus had low collinearity. Therefore, it was included in the model. The term was an interaction between journals listed in the DOAJ and the publishers being members of COPE. This interaction term was included in a multivariate logistic regression model. The regression models including and excluding the interaction term differed as per the Chunk test [P-value: <0.001]. Thus, we selected the model including the interaction terms for further analysis since there was a significant interaction term within our model.

The model also showed that the journal presence on the ISSN portal significantly increased the odds of inclusion in Scopus by 5.451 [AOR=5.451; ME=0.182; p-value <0.001]. The effect of COPE membership on the journal’s inclusion in Scopus varied depending on whether the journal was listed in the DOAJ. Journals listed in the DOAJ but whose publishers are COPE members have significantly reduced odds of being included in Scopus (about 16% of the odds compared to those without this combination); AOR=0.161; ME=-0.196] as shown in Table 5. The logistic regression with interaction terms showed that when journals are listed in the DOAJ and their publishers are members of COPE, there is a significant interaction that leads to reduced inclusion in Scopus [P-value: <0.01]. OA, journal listing on the INASP platform, and publisher’s geographic location were insignificant predictors of inclusion in Scopus.

**Table 5.** Results from logistic regression of factors associated with journal discoverability in Scopus.

Variables	AOR	Robust Standard errors	Sig ^1^	Average Marginal Effects (ME)	Sig ^1^
OA journal	0.766	0.190	n/s	-0.029	n/s
Journals listed in DOAJ	6.873	2.072	***	0.207	***
Journals present on the ISSN portal	5.451	2.069	***	0.182	***
The journals whose publisher was a member of COPE	10.496	3.340	***	0.253	***
Journals present on the INASP listing	1.139	0.236	n/s	0.014	n/s
Publisher based in Africa	0.919	0.302	n/s	-0.009	n/s
Interaction between OA journals listed in the DOAJ and the journals' publisher being members of COPE	**0.161**	**0.086**	**	-0.196	**
Constant	0.027	0.013	***		
Number of Observations	1116				
Pseudo R ^2^	0.22				

OA: Open access; NOA: Not open access; DOAJ: Directorate of Open Access Journals; ISSN: International Standard Serial Number; COPE: Committee on Publication Ethics; INASP: International Network for Advancing Science and Policy; AOR: Adjusted Odds Ratio.

^1^ Statistically significant is indicated by level, that is,

* p<0.05,

** p<0.01,

*** p<0.001 and n/s=not significant.

### Multivariate analysis of factors associated with the discoverability of journals by Google Scholar and Inclusion in Scopus

The collinearity test showed that one relevant interaction term associated with the discoverability of journals by Google Scholar and inclusion in Scopus had low collinearity. The term was an interaction between journals listed in the DOAJ and the publisher being a member of COPE. Therefore, it was included in the model and was significant [P-value: <0.001]. The regression models including and excluding the interaction term differed as per the Chunk test [P-value: <0.001]. Thus, we selected the model including the interaction term for further analysis since the interaction term within the model was significant.

The model showed that the journal’s presence on the ISSN portal significantly increased the odds of being discoverable by Google Scholar and inclusion in Scopus by 4.999 [AOR=4.999; ME=0.134; p-value <0.001]. The effect of DOAJ listing on the journal’s discoverability by Google Scholar and inclusion in Scopus varied depending on whether the journal’s publisher was a COPE member. Journals listed in the DOAJ but whose publishers are COPE members have significantly reduced odds of being discoverable by Google Scholar and being included in Scopus (about 11.6% of the odds compared to those without this combination); AOR=0.116; ME=-0.180] as shown in Table 6. The logistic regression with interaction terms showed that when journals are listed in the DOAJ and their publishers are members of COPE, there is a significant interaction that leads to reduced inclusion in Scopus [P-value: <0.01]. OA, journal listing on the INASP platform, and publisher’s geographic location were insignificant predictors of discoverability by Google Scholar and inclusion in Scopus.

**Table 6.** Results from logistic regression of factors associated with journal discoverability by Google Scholar and inclusion in Scopus.

Variables	AOR	Robust Standard errors	Sig ^1^	Average Marginal Effects (ME)	Sig ^1^
OA journal	0.802	0.238	n/s	-0.018	n/s
Journals listed in DOAJ	8.816	2.950	***	0.182	***
Journals present on the ISSN portal	4.999	2.376	**	0.134	**
Journals whose publisher was a member of COPE	9.458	3.256	***	0.188	***
Journals present on the INASP listing	1.302	0.312	n/s	0.022	n/s
Publisher based in Africa	0.905	0.310	n/s	-0.008	n/s
Interaction between OA journals listed in the DOAJ and the publisher being members of COPE	**0.116**	**0.064**	***	-0.180	***
Constant	0.015	0.009	***		
Number of Observations	1116				
Pseudo R ^2^	0.21				

OA: Open access; NOA: Not open access; DOAJ: Directorate of Open Access Journals; ISSN: International Standard Serial Number; COPE: Committee on Publication Ethics; INASP: International Network for Advancing Science and Policy; AOR: Adjusted Odds Ratio.

^1^ Statistically significant is indicated by level, that is,

* p<0.05,

** p<0.01,

*** p<0.001 and n/s=not significant.

### Multivariate analysis of factors associated with the discoverability/inclusion of journals on at least one platform (Google Scholar and Scopus)

Relevant interaction terms with low collinearity for factors associated with the discoverability/inclusion of journals in at least one platform (Google Scholar and Scopus) were introduced in a multivariate logistic regression model. Results from the Chunk test indicated that there were insignificant interaction terms within the model [P-value = 0.294]. The estimation results of the logistic regression model without interaction terms are presented in Table 7. Results of the logistic regression showed that three factors significantly increased the journal discoverability/inclusion in at least one platform (Google Scholar or Scopus). The odds of being included and discoverable on at least one platform are 3.757 times larger for OA journals listed in DOAJ compared to those that are not [AOR=3.757; ME=0.261; p-value<0.001]. Being present on the ISSN portal increased the odds of being discoverable and included in at least one platform by 2.383 [AOR=2.383; ME=0.171; p-value<0.01] and having a publisher who is a member of COPE increased the odds of being discoverable and included in at least one platform by 5.412 [AOR=5.412; ME=0.333; p-value<0.001].

**Table 7.** Results from logistic and probit regression of factors associated with journal discoverability/inclusion in at least one platform (Google Scholar and Scopus).

Variables	AOR	Robust Standard errors	Sig ^1^	Average Marginal Effects (ME)	Sig ^1^
OA journal	0.947	0.155	n/s	-0.011	n/s
OA journals listed in DOAJ	3.757	0.955	***	0.261	***
Journals present on the ISSN portal	2.383	0.415	***	0.171	***
Journals whose publisher was a member of COPE	5.412	1.643	***	0.333	***
Journals present on the INASP listing	0.930	0.138	n/s	-0.014	n/s
Publisher based in Africa	0.830	0.271	n/s	-0.037	n/s
Constant	0.269	0.098	***		
Number of Observations	1116				
Pseudo R ^2^	0.12				

OA: Open access; NOA: Not open access; DOAJ: Directorate of Open Access Journals; ISSN: International Standard Serial Number; COPE: Committee on Publication Ethics; INASP: International Network for Advancing Science and Policy; AOR: Adjusted Odds Ratio.

^1^ Statistically significant is indicated by level, that is,

* p<0.05,

** p<0.01,

*** p<0.001 and n/s=not significant.

Being an OA journal [AOR=0.947; ME=-0.011], being present on the INASP listing [AOR=0.930; ME=-0.014], and having a publisher based in Africa [AOR=0.830; ME=-0.037] decreased the odds that a journal would be discoverable on at least one platform, but these associations were insignificant.

## Discussion

This study focused on characterizing the various factors associated with the discoverability of African research work by Google Scholar and inclusion in Scopus. African journals were nearly four times more likely to be discovered on Google Scholar than being included in Scopus. These findings concur with a study done which found that Google Scholar consistently includes a large percentage of citations across all thematic areas (93%-96%), ahead of Scopus (35%-77%), and finds nearly all the Scopus (92%) citations. ^22^ Our findings indicated there were three main predictors of African journals on at least one of these two platforms, that is, (i) the listing of the OA journal in DOAJ, (ii) the presence of the journal on the ISSN portal, and (iii) the membership of the journal’s publisher on COPE. This information is crucial for strategizing the editorial process of journals in Africa with an aim of higher discoverability and inclusion of the research that they publish. Furthermore, African researchers can make informed decisions about which journals are best suited to maximize the visibility of their research outputs.

Our finding that African journals generally have lower discoverability by Google Scholar and inclusion in Scopus has been outlined in previous studies ^42^^,^^43^ which showed that journals in Africa were less discoverable than their international counterparts and this was due in part to constrained financial resources and reduced submissions by African authors who prefer to publish in international journals. ^43^ Additionally another study conducted on biomedical journals in Sub-Saharan Africa established that, only one quarter was found in at least one of the biomedical databases. ^43^ Interestingly, a journal being OA alone was not a significant predictor of being discoverable by Google Scholar and inclusion in Scopus, and a journal had to be both OA and listed on DOAJ to be discoverable/included in at least one of the platforms. Being OA is necessary but not sufficient to significantly predict discoverability and inclusion. These findings contradict earlier findings which showed that Google Scholar has a higher coverage of OA African journals than NOA journals. ^44^ We found that of the 354 journals that were discoverable by Google Scholar, only 42.4% were OA journals. However, when an OA journal was listed in the DOAJ it was noticeably more discoverable than those that are not. We note that while being OA alone does not improve the journal’s discoverability and inclusion, being listed in DOAJ gives journals an important advantage of being discoverable/included in at least one platform (Google Scholar and Scopus) over those that are not listed. This is in line with DOAJ’s objective to ensure that journals are accessible to as many people as possible. As such African journals should adopt editorial processes that would ensure that they are eligible for listing in DOAJ to increase the journals’ discoverability and access to their research content.

The presence of the journal on the ISSN portal was a significant predictor of the journal being included and discoverable by Google Scholar, Scopus, and at least one of them. These findings are consistent with a previous systematic study that found that the presence of an ISSN was a significant predictor of journals’ discoverability by Google Scholar. ^44^ Having this ISSN is vital for qualifying for listing on the ISSN portal and African journals should consider adhering to ISSN portal inclusion criteria to harness this discoverability advantage. It is also key to maintain those standards and the subscription to ensure that the journal continues to be listed in the ISSN portal.

For those journals where the publisher of the journal was a member of COPE, the journal was more likely to be included and discovered by at least one platform. As of now and to our knowledge, there are no studies that examined the association between COPE membership of a journal publisher and the journal’s subsequent discoverability and inclusion. This study pioneers the characterization of this association with the recommendation that African journals endeavor to meet the requirements of being members of COPE to increase their discoverability and inclusion in either Google Scholar, Scopus, or both.

Other studies have found that listing on INASP increases journal discoverability and inclusion in databases ^28^ but our findings show that this listing is not important for increasing the chance that a journal will be included and discovered on Google Scholar and/or Scopus. Furthermore, our study shows that having an Africa-based publisher was not likely to significantly affect whether or not a journal was published on these two platforms.

This study established that the effect of DOAJ listing on the journal’s discoverability by Google Scholar and inclusion in Scopus varied depending on whether the journal’s publisher was a COPE member. Journals might face specific challenges or characteristics that reduce discoverability and inclusion (e.g., technical indexing issues, specific policies of Google Scholar, or Scopus). Journal discoverability is not always straightforward and may be influenced by more nuanced factors beyond general quality markers like DOAJ listing or COPE membership. However, there is a need to align the quality markers to align with the specific policies of Google Scholar and Scopus.

There were a few limitations in the implementation of this study. For one, while AJOL and Sabinet list journals from Africa, it is probable that some are not listed and that this may not be random. What’s more, this study is descriptive and was conducted and concluded in July 2023. We acknowledge that a subsequent search of these two platforms might yield different results. Finally, discoverability by Google Scholar and inclusion in Scopus evolve as the curation of one and the algorithms of the other evolve.

In summary, the findings from this study contribute new evidence to the currently small, but growing, evidence on the discoverability and inclusion of African journals on international platforms surrounding the key drivers for poor journal discoverability. This evidence is vital for driving interventions that improve the identified major factors that increase African journals’ discoverability and inclusion in databases. Promoting the journals in academic communities is key and this can be achieved through target marketing and outreach efforts. As a matter of action, a potential area for research would examine the effectiveness of different marketing strategies (targeted email campaigns, paid advertising, and search engine optimization among others). Furthermore, encouraging African researchers to submit their work to African journals and to cite the work published in these journals in their research would build a critical mass of demand that would stimulate journals to evolve to meet this demand. Additionally, it is crucial to foster partnerships between African journals and international publishers and indexing databases to make these journals eligible for these databases. We also explored other factors that may explain the discoverability of African journals on these platforms and found that they were not very meaningful for determining a journal’s discoverability and/or inclusion in the platforms. Specifically, these factors included (i) being an OA journal, (ii) being present on the INASP listing and (iii) having an Africa-based publisher. While these were not important factors for African journal discoverability and inclusion, it is worth considering in future efforts towards such studies.

## Conclusion

Although the discoverability of African journals on Google Scholar and inclusion in Scopus is low compared to non-African journals, improvements of a structural nature are possible with interventions aimed at improving factors that predict discoverability and inclusion. Therefore, programs aimed at improving the discoverability and inclusion of African journals should focus on developing and/or upgrading these journals to ensure they are listed on the ISSN portal. Interventions aimed at ensuring African journals are discovered/included in either Google Scholar, Scopus, or both should focus on making the OA journals listed in DOAJ and ensuring the journals’ publishers are COPE members. Journals might face specific challenges or characteristics that reduce discoverability and inclusion (e.g., technical indexing issues, specific policies of Google Scholar, or Scopus). Journal discoverability is not always straightforward and may be influenced by more nuanced factors beyond general quality markers like DOAJ listing or COPE membership. However, there is a need to align the quality markers to align with the specific policies of Google Scholar and Scopus.

## Data Availability

Zenodo: Factors Affecting African Journals Visibility Data Archive, https://doi.org/10.5281/zenodo.7810935. ^45^
